# Justice implications of health and food security policies for Indigenous peoples facing COVID-19: a qualitative study and policy analysis in Peru

**DOI:** 10.1093/heapol/czad051

**Published:** 2023-11-16

**Authors:** Victoria Chicmana-Zapata, Ingrid Arotoma-Rojas, Cecilia Anza-Ramírez, James Ford, Eranga K Galappaththi, Kerrie Pickering, Emma Sacks, Cecil Togarepi, Chrishma D Perera, Bianca van Bavel, Keith Hyams, Francis A Akugre, Jonathan Nkalubo, Indunil Dharmasiri, Olivia Nakwafila, Adelina Mensah, Jaime J Miranda, Carol Zavaleta-Cortijo

**Affiliations:** Universidad Peruana Cayetano Heredia, Av. Honorio Delgado 430, San Martín de Porres, Lima 15102, Perú; Priestley International Centre for Climate, University of Leeds, Woodhouse, Leeds LS2 9JT, United Kingdom; CRONICAS—Universidad Peruana Cayetano Heredia, Av. Armendáriz 445, Miraflores, Lima, 15074, Perú; Priestley International Centre for Climate, University of Leeds, Woodhouse, Leeds LS2 9JT, United Kingdom; Department of Geography, Virginia Polytechnic Institute and State University, 238 Wallace Hall, Blacksburg, VA 24060, USA; Environmental Sustainability Research Centre, Brock University, 1812 Sir Isaac Brock Way, St. Catharines, ON L2S 3A1, Canada; Johns Hopkins Bloomberg School of Public Health, 615 N Wolfe St., Baltimore, MD 21205, USA; University of Namibia, Ogongo Campus, Private Bag X5507, Eliander Mwatale Street, Oshakati, Namibia; Department of Geography, Virginia Polytechnic Institute and State University, 238 Wallace Hall, Blacksburg, VA 24060, USA; University of Leeds, Woodhouse, Leeds LS2 9JT, United Kingdom; University of Warwick, Coventry CV4 7AL, United Kingdom; Institute for Environment and Sanitation Studies, University of Ghana, MR39+C4X, Annie Jiagge Rd, Accra, Ghana; Mulago National Referral Hospital and Uganda National Health Research Organization, Mulago Hospital Complex, Mulago Hill, P.O. Box 7051, Kampala, Uganda; Department of Geography, Virginia Polytechnic Institute and State University, 238 Wallace Hall, Blacksburg, VA 24060, USA; University of Namibia, 340 Mandume Ndemufayo Avenue, Pioneerspark, Namibia; Institute for Environment and Sanitation Studies, University of Ghana, MR39+C4X, Annie Jiagge Rd, Accra, Ghana; CRONICAS—Universidad Peruana Cayetano Heredia, Av. Honorio Delgado 430, San Martín de Porres, Lima 15102, Perú; Unidad de Ciudadania Intercultural y Salud Indígena (UCISI), Facultad de Salud Pública y Administración, Universidad Peruana Cayetano Heredia, Av. Honorio Delgado 430, San Martín de Porres, Lima 15102, Perú

**Keywords:** Indigenous, justice, COVID-19, health, food

## Abstract

The spread of COVID-19 in Peru resulted in the declaration of a national health emergency, in which Indigenous peoples were identified as being particularly vulnerable due to their pre-existing poor health indicators and disadvantaged social conditions. The aim of this paper is to examine how the Peruvian government responded to the health and food needs of the Shawi and Ashaninka Indigenous peoples of Peru during the first 18 months of the pandemic (March 2020–August 2021). This study uses both official policy documents and real-world experiences to evaluate policy responses in terms of their immediate impact and their longer-term sustainability and contribution to the improvement of health, well-being and justice for Indigenous communities. Four health and food security responses were evaluated: the Amazon Health Plan and Indigenous Command; food aid; cash aid; and COVID-19 vaccination. We employed the Multidimensional Injustice Framework to analyse the justice implications of the design and implementation of responses. Data collection included 71 interviews with government officials (*n* = 7), Indigenous leaders (*n* = 31) and community members (*n* = 33). The results show how national and regional governments released policies to address the health and food needs of Indigenous peoples directly or indirectly, as part of a broader focus on vulnerable people. However, justice implications were not sufficiently addressed in the design or implementation of the responses. On the distributive dimension, Indigenous communities were prioritized to receive health goods and services, nevertheless, the distribution had shortcomings that impeded their collection and Indigenous food systems and livelihoods were largely overlooked. On the procedural dimension, Indigenous representatives were included to provide culturally sensitive feedback on health interventions, but without funding, and furthermore, the community members had only passive participation. This paper points out the importance of considering and addressing justice implications for more effective and fairer health and food policy responses to current and future health crises.

Key messages

- Despite existing policies aimed to assist Indigenous peoples in health and food security during COVID-19, in numerous cases justice dimensions were not adequately addressed.
- Peruvian government policy prioritized Indigenous communities to receive health goods and services but no funds were allocated to the Indigenous representatives’ collaborating in the responses.
- Peruvian government policy did not consider Indigenous food systems and livelihoods, limiting the uptake of resources provided.
- Peruvian government policy included, in some cases, Indigenous representatives to provide culturally sensitive feedback on health interventions, but Indigenous community members participated only as passive beneficiaries.

## Introduction

COVID-19, an infectious disease caused by Severe Acute Respiratory Syndrome Coronavirus 2 (SARS CoV-2), negatively affected health systems worldwide, causing infections, deaths and disrupting health service delivery (Haileamlak, 2021). The virus and the measures to contain its spread had profound implications for food security, widening inequalities and jeopardizing livelihoods, with worst consequences for the most vulnerable (HLPE, 2020).

Peru, a country that already faces health system weaknesses (Gianella *et al.*, 2020), was hit particularly badly by COVID-19. The first case was detected in March 2020, and despite the prompt actions of Peru’s national government, the responses failed to contain the spread of COVID-19 and its consequences. For the years 2021 and 2022, Peru had the highest proportion of excess deaths due to the pandemic in the world, doubling the number of expected deaths (Msemburi *et al.*, 2023).

COVID-19 exposed pre-existing challenges that the Peruvian government was experiencing in responding to health crises: fragmented and unequal health services; deficient emergency response plans; and a poor capacity to deliver public services equitably. The pandemic brought about a worsening of food insecurity in Peru, as well. In 2022 more than half of the Peruvian population was food insecure (FAO *et al.*, 2022).

In Peru, as around the world, Indigenous peoples were among the most vulnerable populations facing COVID-19, due to their poor access to essential services and their nutritional deficiencies (Zavaleta-Cortijo *et al.*, 2020; Ford *et al.*, 2022). Before COVID-19, more than a fifth and a third of Indigenous children under 5 years of age had malnutrition and anaemia, respectively (INEI, 2020). According to the 2017 National Census, only 19% of Amazon Indigenous peoples had access to a public water network at home and 12% to a public sewage system, compared to more than 50% of non-indigenous peoples with access to these services (INEI, 2017). In fact, Amazon Indigenous peoples were among the most infected populations during the COVID-19 pandemic. By mid-2020, they represented 4% of COVID-19 cases at the national level (Dirección de Epidemiología del Ministerio de Salud, 2020), although they account for only 0.9% of the total population (INEI, 2017). Against this background, and considering future health crises, governments need to guarantee health and food security for Indigenous peoples in a manner that fully incorporates concerns about justice and equality. A critical research gap exists on how disaster and health crisis management responses incorporate Indigenous perspectives, knowledge and practices (Bacud, 2017; Ali *et al.*, 2021). The COVID-19 pandemic has also exposed weaknesses in common frameworks to evaluate preparedness for public health crises, considering the role and complexities of social, economic, political and ecological factors (Traore *et al.*, 2023). To fill these gaps, this paper uses a holistic approach, the Multidimensional Injustice Framework (MDIF), to examine how well government COVID-19 responses addressed the health and food needs of Indigenous peoples in Peru. The MDIF recognizes that justice cannot be measured with a single indicator and that analyses of food and health policies need to consider how multiple factors interact to produce inequality (Byskov *et al.*, 2021). The MDIF holds that inclusion, participation and recognition of vulnerable groups are key considerations when planning policies to address the root of the problem, without reproducing existing injustices (Byskov *et al.*, 2021). For the case of Indigenous people, the lack of consultation and recognition of indigenous values in environmental policies, for example, have resulted in increasing socioeconomic marginalization within their countries (da Rocha *et al.*, 2017). Moreover, MDIF aligns well with a justice approach in global health, advocating for pandemic responses that target the ‘needs of those with the most restricted opportunities to be healthy’ (Aldcroft *et al.*, 2023).

Using policy analysis and qualitative research methods, this study evaluates the justice implications of four key health and food security responses in Peru. The results are divided into a policy mapping of responses to assist Indigenous peoples during the first 18 months of the COVID-19 pandemic (March 2020–August 2021), and interviews to understand how well the stated policies were implemented. The results are presented and discussed in relation to two key dimensions of the MDIF, fairness in the distribution of resources (distributive justice) and fairness in the process of distribution (procedural justice). Our results contribute to enhancing the understanding of effective equitable distribution of resources and capabilities necessary to meet the specific needs, perspectives and knowledge systems of Indigenous communities. Our work demonstrates how effective and just governmental policies can support and prioritize the recognition, representation and participation of Indigenous peoples in developing and implementing responses to ongoing and future emergent global crises.

## Methodology

### Conceptual framework

**Figure 1.**
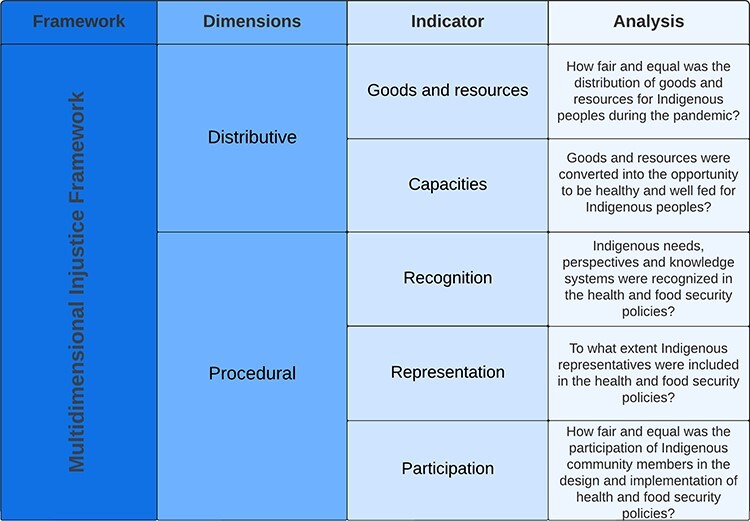
Multidimensional Injustice Framework adopted from Byskov *et al.* (2021) and modified by the authors for this research

As a basis for examining the (in)justice implications of our findings, the four key responses by the Peruvian government were analysed using the MDIF, adapted from Satyal *et al.* (2020) and Byskov *et al.* (2021). The MDIF seeks to understand and tackle issues of injustice and inequity in development contexts. The framework identifies two broad categories of injustice: distributive and procedural. *Distributive justice* reflects the distribution of goods and resources within a society, along with the distribution of capabilities to meet the needs of society members. Indicators of distributive justice include the nature of the distribution of goods and resources and the conversion of the former into capacities. *Procedural justice* refers to the inclusion of peoples’ claims and interests in the development and implementation of policy. The three aspects of procedural justice indicators are (1) recognition: the consideration of needs, perspectives and knowledge systems; (2) representation: the inclusion of community representatives; and (3) participation: involvement of local communities. Participation can take various forms: passive when communities are included only as response receivers; consultative when communities are consulted in the design response; or collaborative when communities provide feedback on the implementation response and have authority that is centred in Indigenous value systems and context (David-Chavez and Gavin, 2018; Coggins *et al.*, 2021). The framework indicators were adapted to be applicable to the analysis of health and food security policy for Indigenous peoples (see Fig. 1).

### Study design

Data were collected as part of the COVID Observatories, a research programme that aims to monitor how COVID-19 interacted with multiple stresses to affect Indigenous peoples, co-generating knowledge and capacity to strengthen resilience in 11 countries worldwide, between January 2021 and March 2023 (COVID Observatories Project, 2022). For this study, we used a qualitative approach, with two components: an analysis of policy responses (both national and regional levels); and interviews with key informants.

### Collaboration with Indigenous populations

**Figure 2.**
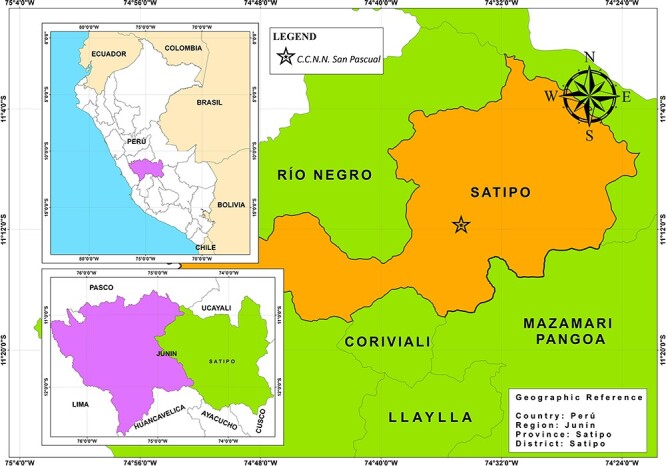
Map of the location of the Ashaninka community participating in this study, made by Engler Puente with ArcGis 10.8 software. Source: National Institute of Statistics and Informatics

**Figure 3.**
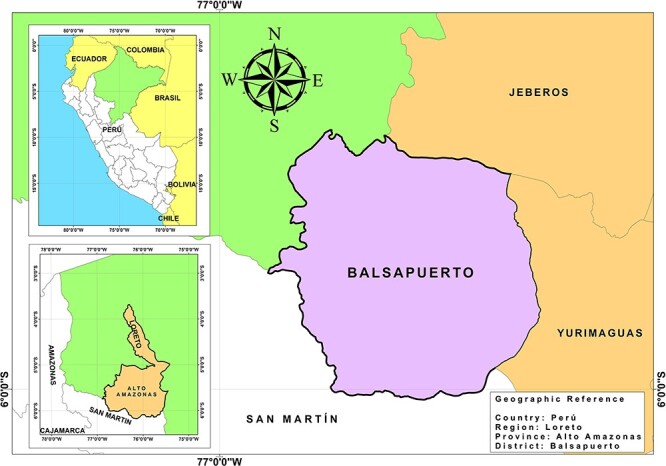
Map of the district of the Shawi community participating in this study, made by Engler Puente with ArcGis 10.8 software. Source: National Institute of Statistics and Informatics

This study focuses on the Ashaninka and Shawi Indigenous peoples, who inhabit the centre and east of the Peruvian Amazon, respectively. The Ashaninka participants in Satipo province, Junín region (Fig. 2), and the Shawi participants in the Alto Amazonas province, Loreto region (Fig. 3). Like other Indigenous peoples in Peru, the Ashaninka and Shawi have limited access to public health services and experience high rates of poverty and social exclusion (Torres-Slimming *et al.*, 2019; Badanta *et al.*, 2020). They have a ‘dual food system’ comprising traditional foods sourced by farming, gathering, fishing, hunting, exchanging or sharing among community members, and non-traditional foods they purchase or receive through social assistance (Gushiken *et al.*, 2011; Zavaleta *et al.*, 2017; 2018; Arotoma-Rojas *et al.*, 2022).

### Data collection

The methods included a search and analysis of policy documents and interviews with key informants. National and regional policy documents for assistance to Indigenous peoples, dating from March 2020 to August 2021, were identified for analysis. The search was undertaken using a Peruvian search-engine of legal norms (El Peruano, 2023). We consider the Peruvian Ministerio de Cultura (2014) definition of ‘policy’ as the set of objectives, decisions and actions of governments to solve important problems of the citizens.

To develop insights into the experience and response to COVID-19 across the pandemic cycle, we repeatedly interviewed ‘Observers’ from February 2021 to June 2022. We first interviewed a total of 18 Observers of three types: Shawi and Ashaninka Indigenous community members (*n* = 6); national and regional Indigenous representatives (*n* = 5); and government officials from the health sector or working with Indigenous peoples (*n* = 7), see Table 1.

**Table 1.** List of key informants

#	Type of informant	Sex	Scope	Sector
01	Indigenous representative	Male	National	Indigenous organization
02	Indigenous representative	Male	Regional	Shawi organization
03	Indigenous community member	Female	Regional	Shawi community
04	Indigenous community member	Female	Regional	Ashaninka community
05	Indigenous representative	Female	National	Indigenous organization
06	Indigenous community member	Female	Regional	Ashaninka community
07	Indigenous community member	Male	Regional	Ashaninka community
08	Indigenous community member	Male	Regional	Shawi community
09	Indigenous community member	Male	Regional	Shawi community
10	Indigenous representative	Male	Regional	Ashaninka organization
11	Indigenous representative	Female	National	Indigenous organization
12	Government official	Male	Regional	Local government
13	Government official	Female	Regional	Health sector
14	Government official	Male	Regional	Health sector
15	Government official	Male	Regional	Regional government
16	Government official	Male	Regional	Culture sector
17	Government official	Male	Regional	Health sector
18	Government official	Female	Regional	Health sector

Each Observer had an in-depth baseline interview about the epidemiology, lived experiences and responses to COVID-19. Then, monthly interviews to follow up on any changes in their observations were continued only with the Indigenous Observers, i.e. community members and representatives. In total, there were 71 interviews, from community members (*n* = 33), Indigenous representatives (*n* = 31) and government officials (*n* = 7). For the period in which baseline and follow-up interviews were conducted with each observer, see Table 2.

**Table 2.** Baseline and follow-up interviews applied to the Observers

	2021											2022						
# Observer	Feb	Mar	Apr	May	Jun	Jul	Aug	Sep	Oct	Nov	Dec	Jan	Feb	Mar	Apr	May	Jun	Jul
1	BL	FU	FU		FU			FU	FU	FU		FU						
2	BL		FU	FU	FU			FU	FU		FU	FU						
3		BL	FU					FU	FU	FU		FU						
4			BL	FU	FU				FU	FU	FU	FU						
5			BL	FU		FU		FU	FU	FU	FU							
6				BL	FU				FU	FU	FU	FU						
7				BL	FU				FU	FU	FU	FU						
8								BL	FU	FU	FU							
9								BL	FU	FU	FU							
10									BL		FU		FU					
11									BL	FU	FU		FU	FU				
12																BL		
13																BL		
14																	BL	
15																	BL	
16																	BL	
17																	BL	
18																		BL

BL, baseline interview; FU, follow-up interview.

Interviews were conducted virtually (by phone or video call) and in person when the Observer solicited it and there were no mobility restrictions. The interview length was 1–2 hours. All interviews were in Spanish; the Indigenous Observers were given the chance to have their interviews in their Indigenous language, but they chose not to. The guide for the baseline and follow-up interviews can be found in Supplementary Material, Appendix A.

### Data analysis

The selection of the government responses was made through three focus groups comprised of four members of the research team (VCH, IAR, CA and CCZ), all women researchers based in Peru, with previous experience of working with Indigenous peoples in the study regions. The focus on health and food security was chosen because it was an important topic that emerged during the initial interviews with the Observers. Then, based on the policy mapping and the interviews, the four response strategies impacting most heavily on the health and food situation of Indigenous populations were selected.

The prioritized responses were the Amazonian Plan and the Indigenous COVID-19 Commands, food aid, cash aid, and the COVID-19 vaccination programme. The information from the interviews and the prioritized policy documents were analysed in Spanish with Nvivo (2019 version), with double coding by three researchers. The coding themes were established a priori, using MDIF indicators: goods and resources; capacities (distributive dimension); and representation; recognition and participation (procedural dimension). Selected quotes from the interviews were translated into English and included in the results to illustrate key findings.

## Results

The results are divided into a policy mapping of responses to assist Indigenous peoples, followed by an analysis of the four key health and food security policies selected. The policy mapping subsection presents a narrative summary of 43 different national and regional responses to assist Indigenous peoples in Peru, during the first 18 months of the pandemic (March 2020–August 2021). In the section on prioritized responses, four key health and food security responses were selected from the policy mapping, and their justice implications analysed using the MDIF.

### Policy mapping: national and regional policy documents

**Figure 4.**
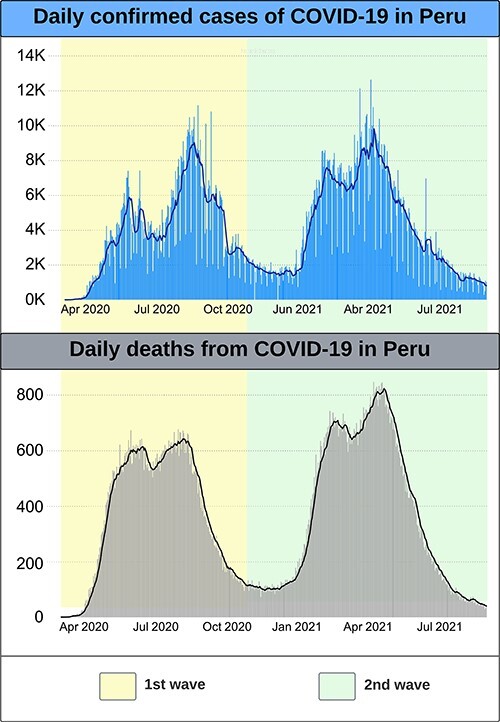
Epidemiological graphics of daily COVID-19 cases and deaths in Peru during the study period (March 2020–August 2021), provided by the co-author Cecilia Anza-Ramirez. Source: Peruvian Ministry of Health

We analysed the 43 national and regional efforts undertaken by the government to assist Indigenous peoples from March 2020 until August 2021, covering two waves of COVID-19 (Fig. 4). The complete list of policies can be found in the Supplementary Material, Appendix B. Policies directed at assisting Indigenous peoples came predominantly from the national government.

During the first wave (March–November 2020), a national quarantine was announced, business, transportation and schools were closed, and mandatory sanitary protocols were implemented. Regional governments provided transportation for Indigenous migrants to return from their temporary employment to their regions of origin, but the governments eventually ran out of capacity and budget. A national government decree established actions to protect Indigenous peoples, including guaranteeing their language rights, providing culturally adequate public services, and protecting Indigenous peoples living in isolation (Gobierno del Perú, 2020c). The national government also created a temporary multisectoral commission, formed by Indigenous organizations, to advise the government on actions for Indigenous peoples (Gobierno del Perú, 2020f).

A specific plan on health interventions was developed for Amazon Indigenous peoples (Ministerio de Salud, 2020a). Further, the Ministry of Culture published information in local languages about COVID-19 and how to avoid catching it, using culturally appropriate examples and explanations. The number of COVID-19 cases in Peru was analysed by ethnicity; however, this did not extend to mortality (Gobierno del Perú, 2020d). In Junín and Loreto, regional governments provided ivermectin to prevent and treat COVID-19 and they included Indigenous peoples in this strategy. Ivermectin is an antiparasitic medicine that was later shown to be ineffective in preventing and treating COVID-19 (Chirinos *et al.*, 2020; Taype-Rondan *et al.*, 2020). Socioeconomic aid was launched to lessen the economic crisis: social programmes were brought forward (Gobierno del Perú, 2020e); aid in the form of cash and food were given to vulnerable populations (Gobierno del Perú, 2020a). Many Indigenous peoples were beneficiaries of these measures since they are identified as vulnerable populations.

During the second wave of COVID-19 (December 2020–October 2021), targeted quarantines were implemented in regions with the most cases of COVID-19, socioeconomic aid continued, and a COVID-19 vaccination programme commenced with measures to promote it.

### Analysis of prioritized health and food policy responses

This section presents the analysis of the justice implications of four prioritized health and food security responses to assist Ashaninka and Shawi Indigenous peoples in Peru during the first 18 months of the COVID-19 pandemic. The findings are structured using the MDIF indicators of distributive justice (goods and resources, and capacities) and procedural justice (recognition, representation and participation) (Table 3).

**Table 3.** Prioritized responses and the Multidimensional Injustice Framework (MDIF)

	Distributive justice indicators		Procedural justice indicators		
Prioritized responses	Goods and resources	Capabilities	Recognition	Representation	Participation
Amazon Health Plan and Indigenous COVID-19 Command	Amazon Health Plan provided resources for COVID-19 prevention and treatment, but no budget for the Indigenous representatives responsible for managing implementation of the plan in the Commands.	The Commands did not strengthen supervisory skills required by the Indigenous representatives.	The participation of Indigenous representatives in the COVID-19 Commands contributed to culturally appropriate health interventions, such as including Indigenous linkages in the health brigades.	The Command included Indigenous representatives who gave feedback on the implementation of the Amazon Health Plan.	Indigenous representatives in the Command felt their participation was only nominal, they were official members, but their ideas and proposals were minimally listened to and poorly incorporated.
External food aid	Food deliveries had shortcomings, such as misdistribution, and lack of sanitary protocols in the distributions.	The deliveries did not promote food security for Indigenous people because they were infrequent and included poorly nutritious or culturally inappropriate food items.	The food preferences of Indigenous people were not part of the deliveries. Other products that the Indigenous people needed for their livelihoods were not included (e.g. matches, bullets and flashlights).	Indigenous officials or organizations were not considered when selecting food items.	The Indigenous peoples were only passive recipients of the food provision, they were not part of the design or implementation of the response.
Cash aid	The cash aid had shortcomings such as Indigenous peoples in need not being informed, and logistical issues that prevented Indigenous people from collecting the aid.	This aid did not considerably strengthen the Indigenous economy and it exposed them to getting infected with COVID-19.	Design of the cash aid did not consider the Indigenous geographical context. The mechanisms for the delivery of aid to remote Indigenous communities did not work and Indigenous people had to travel far to collect the money exposing them to COVID-19.	Indigenous representatives were not included in the design or implementation of this measure.	Indigenous people were only passive recipients of this measure, by collecting the aid.
Vaccination against COVID-19	Indigenous people were a prioritized group for the COVID-19 vaccines but had unequal access to them because of logistics problems.	The official information about COVID-19 vaccines was disseminated late when there was already strong mistrust of the vaccines among Indigenous peoples.	The informative messages about the vaccines were disseminated in Indigenous languages, and the vaccination brigades included Indigenous personnel.	Indigenous representatives were included in the implementation of the vaccination plan, advising about culturally pertinent measures.	Indigenous representatives were included as collaborators in the vaccination programme, but only after several experiences of vaccination brigades being rejected.

### Policy 1: Amazonian Plan and Indigenous COVID-19 Command

In May 2020, after two months of national quarantine, the central government launched a health intervention plan for Indigenous and rural populations of the Amazon (Ministerio de Salud, 2020a). This plan established the improvement of human resources and medical equipment to prevent and treat COVID-19. In June 2020, the national government established the formation of regional Indigenous COVID-19 Commands, led by Indigenous leaders and regional government officials, to supervise implementation of the Amazonian Plan in Indigenous communities (Ministerio de Salud, 2020b). In Loreto, the Indigenous Command was established in June 2020 (Gobierno Regional de Loreto, 2020), and in Junín, in August 2020 (Gobierno Regional de Junín, 2020).

#### Distributive justice (goods and resources, and capacities)

Despite the Amazonian Plan providing additional resources for prevention and testing of COVID-19, Indigenous leaders in the Commands had no funding to monitor implementation of the Plan. Government Observers stated that the Plan assisted Indigenous peoples of the Junín and Loreto regions by providing trained health personnel, biosafety equipment, fuel and motors for transportation (boats and vehicles), supplies for health community promoters, oxygen balloons, and temporary accommodation for patients with COVID-19.

Health brigades visited Indigenous communities to carry out COVID-19 testing and gave assistance to patients with COVID-19. After months without access to primary health care, the brigades also delivered key services to Indigenous populations for the detection of anaemia, malnutrition and tuberculosis, and antenatal care to pregnant women.

*It had been months, that they [health services] have not really been working or carrying out health care or consultations, the brigades had to take advantage, let’s see, if there are pregnant women, if there was anaemia, that is...take advantages of the opportunity we had to have personnel and that they are entering to the indigenous territories*. (Health sector official)

These resources and services were highly valuable for Junín and Loreto local health providers because they were not usually available, which is indicative of broader issues of distributive injustice across these regions. However, some products were inappropriate or faulty. Fabric masks were considered to be too ‘suffocating’ in the hot weather of the Amazon, and some thermometers and pulse oximeters stopped working after having been used a couple of times.

Indigenous Observers also expressed concern about financial irregularities in the Health Plan. In one of the regions, one Observer claimed medical and transportation supplies were not acquired:

*We do not know where the 65 boat motors for the Amazon are, where they have been distributed, because within the implementation of the plan was to improve the infrastructure of the health care or provide first-aid kits. It has not been seen, the money has disappeared again, and those oxygen balloons and motors have not arrived.* (National Indigenous representative)

Although the Amazonian Plan had a budget, the Indigenous representatives in the COVID-19 Commands were not reimbursed for their time and input including attending virtual meetings and transport to and from Indigenous communities. Additionally, Indigenous representatives lacked the opportunity to build capacity through the Commands. Areas where capacity could have been strengthened included managing virtual meetings platforms, supervisory skills to evaluate the implementation of financial measures, and training on COVID-19 preventive measures among Indigenous communities.

Regardless of the drawbacks for Indigenous representatives, they actively participated in the Commands and gave culturally appropriate contributions. In Loreto, the Command suggested the incorporation of an ‘Indigenous linkage’, a person with health training and knowledge of Indigenous culture and language. This person would be a member of the health brigades and visit Indigenous communities to monitor the COVID-19 situation.

After the successful experience in Loreto, these ‘linkages’ were applied in other regions, such as Junín:

*It was very appropriate to propose the Indigenous linkage because otherwise the health team does not have the expertise of providing a service in the Indigenous language. They tell you to take the contraceptive regardless of what contraceptives are traditionally available according to Indigenous culture. The health team doesn’t do that, but the Indigenous linkage must do it.* (Health sector official)

#### Procedural justice (recognition, representation and participation)

Indigenous people were participated in this policy through the involvement of their representatives. However, our Indigenous Observers pointed out that there were significant deficits in their participation. The Amazon Health Plan and the Indigenous COVID-19 Commands were the result of the advocacy efforts of Indigenous organizations, not a government initiative. The Health Plan was designed using a Western medicine approach, but Indigenous participants collaborated in its implementation, achieving interventions that recognized Indigenous practices, as the Indigenous linkages. In Junín and Loreto Commands, more than a third of the members were Indigenous representatives.

Our government Observers noted that Indigenous representatives had an active role in the Commands, their opinions were considered and respected, and they had adequate dialogue with state officials. However, the Indigenous Observers reported that their participation was only nominal, as they were official members, but their ideas and proposals were rarely listened to and poorly incorporated into the Commands:

*The COVID Command, we’ve heard it, we were talking so much about the COVID Command and then what happen, nothing happened, they were in silent, ‘we are going to create [the Commands] so they [Indigenous peoples] be satisfied with a paper work [the design of the policy but not necessarily its effective implementation]*. (National Indigenous representative)

The inclusion of Indigenous participation was limited to representatives at the regional level. Community members, health promoters and community chiefs were not considered in these responses.

### Policy 2: External food aid

The Peruvian government gave external food aid to vulnerable populations during the COVID-19 pandemic, which included Indigenous peoples. The food programme for school children widened its target group to vulnerable populations^1^ (Gobierno del Perú, 2020b) and local governments were allocated a budget to acquire and distribute food to vulnerable families (Gobierno del Perú, 2020a).

Government guidelines for food aid in the context of COVID-19 established consideration of eating habits for food selection and implementation of sanitary protocols to avoid transmitting COVID-19 during distribution (Presidencia del Consejo de Ministros, 2020). The Ministry of Culture launched an extra guide for the delivery of food in Indigenous communities, highlighting the importance of culturally appropriate approaches such as working with communal authorities and ensuring Indigenous people are protected from discrimination (Ministerio de Cultura, 2020).

#### Distributive justice

Our Observers reported several problems with the delivery of food aid in Indigenous communities in the Junín and Loreto regions. First, Observers in the community of Ashaninka noted that Indigenous families did not always qualify for the aid because according to social registers they were not considered vulnerable population. Second, the Ashaninka and Shawi community of our Observers did not receive the food aid when they required it the most, during the first wave, given the limited access to markets. Our government Observers explained that the reason for the delays in the delivery of food was a lack of accurate data in government social registers about the number and location of Indigenous people. Third, Indigenous representatives reported the misdistribution of food aid including municipality workers (who received a higher salary than many other community members) receiving food deliveries. Fourth, the distribution of food with expired dates was reported in the Loreto region. Fifth, our Observers reported that some distributions to Indigenous communities in the Loreto region during the first wave caused COVID-19 infections because sanitary protocols were not implemented by the local government workers delivering the food. In another region, communities threw away the food because they were fearful of becoming infected with COVID-19.

*[T]he municipalities that arrived with food, to the communities, and in Loreto region, the deliveries carried Covid with the crew of the boat. So, when observing this, the other communities closed their territories and did not even allow us to enter with the health brigades*. (Health government official)

Shortcomings in food aid, along with chronic poor nutritional conditions, prevented Indigenous peoples benefiting from the policy and maintaining good nutrition during the COVID-19 pandemic.

From a distributive justice perspective, even the most basic health care needs were not met. While our Ashaninka and Shawi Observers noted that the food aid provided an important source of nutrition, deliveries were only made once and this was insufficient to sustain their feeding through the first 18 months of the pandemic.

*We needed food, but no support came […] there was none of the food […] We wanted to feed better in the pandemic, but we did not have food in our communities, and we were not given food.* (Shawi community member)

Indigenous Observers were critical of these types of government responses since they provided basic goods and services but did not develop the capacity of Indigenous peoples to achieve sustainable livelihoods. Locally driven initiatives to support the nutritional health of Indigenous peoples were preferred, such as providing seeds to complement Indigenous farms, and training on the preparation of nutritious meals using local food.

#### Procedural justice

The food aid complemented Indigenous food systems in a limited way^2^, despite government guidelines calling for a culturally appropriate approach. The deliveries included food^3^ such as non-Amazonian types of beans and diary products^4^, which despite being nutritious are not commonly consumed by remote Indigenous communities who did not know how to prepare them. Consequently, some Indigenous families threw away these food items.

*[T]hey receive lentils or dairy products, and they throw them away […] They don’t like beans, they say ‘no, I already disliked these beans because they are lousy, horrible’*. (Health government official)

*[T]hey give you, some beans that we don’t know about. For example, the chickpea. The people here receive it, but nobody eats it. They receive I don’t know what beans are, they look like wheat. People here don’t eat wheat. They receive it but do not eat it. Either they throw it away or they give it to the hen because they are not used to it. We have our own food*. (National Indigenous representative)

Conversely, the deliveries included only limited protein sources albeit limited, only tuna and beans. Indigenous peoples often face challenges in obtaining protein-rich food in enough quantities, so its delivery was crucial during the health emergency (Zavaleta *et al.*, 2017; Arotoma-Rojas *et al.*, 2022). Supplies needed to prepare the food were not provided including matches for cooking, bullets, flashlights and batteries for hunting. Non-essential products (e.g. coffee) and processed foods with low nutritional content (e.g. instant soup) were also included.

Government Observers in Junín and Loreto agreed that national food programmes needed to make a greater effort to include Indigenous diets and preferences. Before the pandemic, pertinent cultural foods were trialled for incorporation into food deliveries. The rapid response to assist the population during the COVID-19 pandemic forced government officials to use what was available, overlooking cultural food preferences.

Indigenous peoples were passive recipients of this policy because they only received the food and were not part of developing and implementing the response, nor were they involved in prioritizing beneficiaries or choosing the products to be purchased. Beneficiaries of the food aid were chosen according to the government social register and the products were chosen by local government officials. A considerable gap remained between on-the-ground reality and the ideal of procedural justice.

### Policy 3: Cash aid

During the first and second waves, the central government gave cash aid of between $100 and $200 to vulnerable households^5^, to mitigate the economic impacts of COVID-19. Information for the beneficiaries on how to collect the money was shared in the mainstream media, such as radio, TV and social media. The cash aid could be collected at ATMs, banks, or delivered by the public service boats or security transport companies. A complete list of the cash aid payments that were available is documented in Appendix A.

#### Distributive justice indicators

Distribution of the cash aid had several shortcomings that impeded its collection by Indigenous peoples. First, much of the Indigenous population in need were not beneficiaries, e.g. distribution only occurred in the more densely populated parts of the Ashaninka community because remote houses were not registered in the social records. Second, our Observers reported that Indigenous beneficiaries faced several difficulties in collecting the aid. In both the Ashaninka and Shawi communities, during the first wave of COVID-19, community members were not keen to go outside to collect the aid because of fear of infection. In the second wave, in the Shawi community, the elderly were still afraid of being infected and did not collect the aid.

Third, Indigenous populations did not receive clear information about who were the intended beneficiaries, and how to collect the money. Instructions were published on the internet and in the media, to which the Indigenous population have limited access. In the Shawi community, there was confusion about where to collect the aid, and some made unnecessary trips to the closest city. Similarly, in Satipo, there were people asking for money who had not received instructions on how to collect the aid.

Fourth, there were delays in providing the cash to Indigenous peoples at city banks or in delivering the cash to communities. In the Loreto region, during the first wave, our Observers recalled Indigenous people going to the nearest city to receive their cash aid, but their requests were not dealt with even within 15 days. Similarly, during the second wave in Loreto, the boats that brought cash aid and public services to remote Indigenous communities were delayed. Since the boats did not arrive, community members went to the cities, but the banks did not give them the money either.

These issues caused Indigenous populations, with already precarious livelihoods, to spend their money needlessly and without receiving any the cash aid.

*It is very easy for the people who are in charge, but for the Indigenous people who come from their communities in a two-day trip [to collect their cash aids], rowing in a little canoe, borrowing a boat engine, borrowing a boat, borrowing gasoline, and how they are going to survive the days they are in the city…* . (National Indigenous representative)

Our Indigenous Observers were very critical of this measure because it was perceived as creating dependence and not contributing to developing community capacity or strengthening livelihoods. They state their Indigenous organizations prefer measures to support their economy, with autonomy, fair prices for their products and services, and guaranteed protection of the territory.

#### Procedural justice indicators

The delivery of cash aid did not take account of the geographical remoteness of Indigenous communities. Despite mechanisms for delivering cash aid to remote communities (public service boats or securities transport companies), Shawi and Ashaninka Observers reported that most people had to go to the city to collect the money, exposing themselves to COVID-19 infection.

During the first wave, in the Loreto and Junín regions, urban and rural vulnerable populations were agglomerated resulting in huge queues at banks, which discouraged people from collecting the cash aid. Consequently, Indigenous people who came from the communities to the cities were at increased risk of infection by COVID-19 and of later infecting their communities on their return. One Indigenous Observer recalled their experience in Iquitos city, located in Loreto region:

*[I]t had been seen people around the banks, they would spend the night there, no matter the night, no matter the rain … they would just wait in line.* (National Indigenous representative).

Indigenous peoples were passive recipients of this measure, as they were involved only in collecting the aid. No Indigenous representatives or community members were included in designing or implementing this measure. There was no coordination with community authorities to spread information to beneficiaries about collecting the aid, especially information aimed at the Indigenous population. For implementing deliveries, the permission of communal authorities was supposed to have been sought, but Indigenous organizations complained about deliveries without proper permission (Defensoría del Pueblo, 2020).

### Policy 4: Vaccination against COVID-19

The national vaccination plan was approved in October 2020, with three priority groups; Indigenous peoples being the second (Ministerio de Salud, 2020b). Vaccination efforts started in February 2021 for medical personnel and in March 2021 for elderly persons over 60 years followed by various vaccination shifts according to age. Vaccination of Indigenous people over 18 years began in June 2021, and was implemented by health brigades visiting their communities. The Ashaninka community of our participants was visited once by the vaccination brigades (October 2021) and the Shawi community was visited three times (July, September and November 2021).

#### Distributive justice indicators

Despite Indigenous populations being a priority group in the vaccination plan, logistical issues and a lack of information prevented some Indigenous people—especially those in remote locations—from receiving the COVID-19 vaccines promptly. Up to January 2022, more than six months after vaccination of Indigenous people had started, coverage of the second dose in the districts of our Indigenous Observers was below the national average (78.3%); 64.1% in the Ashaninka district, and 20.8% in the Shawi district (Ministerio de Salud, 2022).

Our Observers reported vaccination brigades were either seriously delayed or never arrived in the Loreto and Junín regions. By 2022, in Loreto, vaccination brigades has not reached the most remote Indigenous communities, and delivery of the booster dose was delayed for several communities. In the Shawi community of our participants, the booster dose arrived a month later than expected, and without proper communication, so some people were far away on their farms and could not receive the vaccine. In the Ashaninka community, the vaccination brigade visited them once but due to low take-up (only one person got vaccinated), the team did not return for the booster dose. For the community, vaccines were available at the closest health post, but for many these were some distance away and the cost of transport was prohibitive. These logistical issues caused Indigenous people to feel cheated, which affected their acceptability of COVID-19 vaccines.

Another limitation of the vaccination plan was that official information was disseminated late. Since the start of the vaccination programme, our Indigenous Observers stated that social media and religious groups spread rumours that vaccination causes sterilization, imprints the seal of the devil, or inserts a chip in the body for control. During this first stage, no reliable and clear information about the vaccines was disseminated to Indigenous populations. Our Ashaninka Observer reports the need for information:

*[T]here is no entity that informs us how these vaccines work because sometimes the population of our community say ‘the vaccine is the same virus, I don’t have a virus, they are going to give me a virus’ sometimes there is a lack of information, we want to know what the information will be or which entity can give us more information*. (Ashaninka community member)

Official dissemination of information about COVID-19 vaccines started simultaneously with the vaccination of Indigenous peoples, in June 2021. At that time, there was already strong opposition to the COVID-19 vaccines among Indigenous peoples. Nevertheless, the information campaigns had a positive effect on gaining acceptance of the vaccines:

*A lot of people are now going [to the vaccination centres], I feel that it is already quite a result of a reflection, and awareness of the population. At first for me, I also seemed quite worrying but this last week it has been seen for that people go to get their booster dose, and when the brigade arrives, the people in the communities receive very well in coordination with the authorities. It seems that the work we have had in that field gave results.* (Shawi Indigenous representative).

#### Procedural justice indicators

The government incorporated Indigenous perspectives in the measures to promote vaccination. The Ministry of Culture produced materials (posters, videos and audio) about the vaccines that were adapted to the cultural context, using for example Indigenous languages, images, music and people. In addition, vaccination brigades hired Indigenous linkages^6^ to provide information in their language, a measure recommended by Indigenous representatives. Our Indigenous Observers noted that information produced by Indigenous people or with Indigenous references was considered more trustworthy than campaigns aimed at the general population, or information given by general health personnel.

In the Shawi community of our Observers, the second visit by the vaccine brigades achieved better acceptance than the first visit, because it included an Indigenous linkage, and the participation of communal authorities.

*[B]efore they go to vaccinate, the technicians have to firstly, to do talks. Now they have no longer gone directly with the vaccines… They went a day before to give talks to all the community, they had an hour of explanation, and in that explanation, I think there was a man who translated… and he could make people understand, because in the community they don’t understand Spanish so much*. (Shawi community member)

Indigenous peoples played a consultative role in this policy because their representatives gave feedback on implementing the vaccine programme in Indigenous communities. At the beginning of the vaccination efforts, there was no coordination with Indigenous representatives. After several vaccination brigades were rejected, health officials decided to work with Indigenous representatives on collaborative strategies to promote vaccination.

The Indigenous representatives advised putting more emphasis on the provision of information and engaging Indigenous community leaders to achieve better acceptance of the vaccines.

*I would like the Health institutions to make meetings with the leaders, promoters and health agents to raise awareness about the vaccines in every community, each community has a health promoter and they must be trained to raise awareness. When the brigade arrives, they want to share information about the vaccines, they want to give 2 hours of talks but sometimes in two hours you won’t be able to convince a lot of people who don’t know about the vaccines*. (National Indigenous representative)

## Discussion

The MDIF utilized in our study provided a holistic approach to assess fairness for Indigenous populations in the Peruvian government responses to COVID-19. Our results show that, despite existing policies aimed at assisting Indigenous peoples in health and food security during the COVID-19 pandemic, in numerous cases, justice dimensions were addressed with a significant shortfall.

On the distributive justice dimension, Indigenous communities were prioritized to receive health goods and services but no funds were allocated to enable Indigenous representatives to collaborate in the responses. Corruption also prevented a just distribution of goods and resources, which has previously been reported as a challenge to implementing health measures in Peru (Bressan *et al.*, 2022). On the procedural justice dimension, Indigenous representatives provided culturally sensitive feedback on health interventions, but Indigenous community members participated only as passive beneficiaries and Indigenous food systems and livelihoods were not taken into consideration, resulting in the resources provided not being utilized.

Achieving distributive justice would require provision of goods and resources to the citizens involved in supervision of the policy, as well as addressing corruption. Procedural justice would require a much more participatory approach, which in turn could be expected to lead to better distributive justice outcomes. This qualitative study shows that weak participation by Indigenous representatives and community members, and lack of recognition of their world views, prevented health and food security policy from being effective in responding to a global emergency. Multidimensional justice indicators can inform more effective policies for vulnerable populations in future pandemics and contribute to stronger global health.

Peruvian government interventions with Indigenous communities have been previously criticized for overlooking local food systems and health practices, resulting in limited improvement of health outcomes, and the potential to increase inequities between Indigenous and non-Indigenous communities (Zavaleta *et al.*, 2017; Correa Astete, 2021; Montag *et al.*, 2021). Similarly, studies of Indigenous peoples around the world show the importance of including Indigenous populations’ perspectives, knowledge and practices in health care responses to disasters (Ford, 2012; Bacud, 2017; Pelzang *et al.*, 2017; Mosurska *et al.*, 2022).

Other case studies using the MDIF have demonstrated how connected injustices experienced by Indigenous peoples undermine the effectiveness of policies to assist these populations to adapt to emergencies and global hazards (Satyal *et al.*, 2020; Galappaththi *et al.*, 2023). Effective policy requires Indigenous peoples to be engaged in decision-making, and to benefit from fair resource distribution (Camargo and Vázquez-Maguirre, 2020) with respect to their institutions and knowledge (Wook, 2019). This research complements and adds new findings on the needs of Indigenous communities and highlights the call for more just and effective government responses to assist them in the event of future health emergencies.

Limitations of the research include a lack of quantitative evidence on the impact of the health and food security situation among the Indigenous populations. Further research evaluating how government responses impacted the health and food security of Indigenous peoples using standard epidemiological indicators is recommended. In addition, even though the study included the opinions and perceptions of Indigenous leaders and community members through the interviews, an Indigenous approach of justice in relation to health and food security was not developed. Thus, we recommend further studies to recognize notions of justice and equity among Indigenous populations. Strengths of the study are the diversity of key informants (Indigenous community members and representatives, and government officials), which affords a more complete approach to understanding the design, implementation and results of the government responses. The study also collected responses over a series of interviews, which gathered information on changes over time.

This study used both official policy documents and real-world experiences to evaluate policy responses, in terms of their immediate impact and their longer-term sustainability and contribution to the improvement of health, well-being and justice for Indigenous communities. As very few health studies include the perspective of Indigenous communities and their representatives, this study adds critical information on the success of various government responses in Peru and recommendations for future health emergencies. Furthermore, our findings contribute to discussions on the cultural safety of current food and health policies that aim to benefit Indigenous communities.

## Conclusions

In Peru, the government launched health and food security initiatives to assist Indigenous people during the health emergency of the COVID-19 pandemic. This paper examined four prioritized responses, which aimed to provide services and goods (health equipment and services, food, cash and vaccines) directly or indirectly to Indigenous people, to ensure their healthy nutritional status. Despite the Peruvian government’s efforts, Indigenous needs still require greater recognition and to be addressed in a fairer and multidimensional way. The results indicate that providing fair assistance to Indigenous populations during disease outbreaks requires the inclusion and financing of their representatives, provision of culturally appropriate goods and services, engaging community participation, and promoting capacities in Indigenous peoples to achieve sustainable responses. Policies to address crises should strive not only to meet the immediate needs of the population but to do so in ways that increase participation and justice.

## Supplementary Material

czad051_Supp

## Footnotes

1. Poor, elderly, disabled, Indigenous population and inmates.

2. Composed of food from the farms (through agriculture), forest (hunting, gathering and fishing) and external food (bought or given).

3. Food preferred by Indigenous people is local food, from their farms, forests and rivers (Zavaleta *et al.*, 2017; Arotoma-Rojas *et al.*, 2022).

4. Dairy is discouraged for the Amazon population, according to the guide for Indigenous food aid (Ministerio de Cultura, 2020).

5. Including poor, beneficiaries of social programmes, people without a formal job and those in rural populations. Households of Indigenous peoples of the Amazon are considered to be in the category of extremely poor.

6. The Indigenous linkages were Indigenous people with health training and knowledge of Indigenous culture and language, already implemented for the Amazon Health Plan.

## Reflexivity Statement

This paper includes authors from diverse regions, Africa, North and South America, and Europe. The leading author and half of the co-authors are based in low- and middle-income countries (LMIC), Peru, Namibia, Uganda and Ghana. More than half of the authors are women, and early career researchers are also included. The lead author is an early career woman from a middle-income country.
