# Correction to: Justice implications of health and food security policies for Indigenous peoples facing COVID-19: a qualitative study and policy analysis in Peru

**DOI:** 10.1093/heapol/czae014

**Published:** 2024-03-04

This is a correction to: Victoria Chicmana-Zapata, Ingrid Arotoma-Rojas, Cecilia Anza-Ramírez, James Ford, Eranga K Galappaththi, Kerrie Pickering, Emma Sacks, Cecil Togarepi, Chrishma D Perera, Bianca van Bavel, Keith Hyams, Francis A Akugre, Jonathan Nkalubo, Indunil Dharmasiri, Olivia Nakwafila, Adelina Mensah, Jaime J Miranda, Carol Zavaleta-Cortijo, COVID Observatory, Justice implications of health and food security policies for Indigenous peoples facing COVID-19: a qualitative study and policy analysis in Peru, Health Policy and Planning, Volume 38, Issue Supplement_2, November 2023, Pages ii36–ii50, https://doi.org/10.1093/heapol/czad051

The originally published version of this manuscript required correction.

In the Introduction section, the following sentence incorrectly attributed quoted text to ‘Aldcroft A, Branch-Hollis A, Phillips T, Sands R. 2023. Editorial: BMJ Public Health: a new public health journal from BMJ. *BMJ Public Health* 1: e000008’:

Moreover, MDIF aligns well with a justice approach in global health, advocating for pandemic responses that target the ‘needs of those with the most restricted opportunities to be healthy’ (Aldcroft et al., 2023).

The sentence should have cited the following article instead:

Khosla R, Venkatapuram S. What is a justice-oriented approach to global health? *BMJ Global Health* 2023;**8**:e012155

This in-text citation and the manuscript’s reference list have been corrected.

