# Supplementary material for: Justice implications of health and food security policies for Indigenous peoples facing COVID-19: a qualitative study and policy analysis in Peru
Source: Health Policy Plan. 2023 Nov 16;38(Suppl 2):ii36–50. doi: 10.1093/heapol/czad051 (PMC10680992; doi:10.1093/heapol/czad051)
Supplement: czad051_Supp [file czad051_supp.zip › suppl_data/Supplementary material _ final_VCH.docx]

**Appendix A: Baseline and follow-up interview guides**

1. **Guide of the Policy Observer Baseline interview**

Metadata

For each observer interview document their:

- Age
- Gender
- Official role in decision making (e.g. community leader, policy adviser in a specific ministry, working with Indigenous rights, etc)
- Jurisdiction in their official role
  - Health, food systems, poverty alleviation ……
  - Scale: national, regional, local, community
  - Indigenous peoples focus in their role (e.g. explicit focus on IPs, …..) / study region focus
  - Role in responding to COVID
- Length of time in position

Theme 1: Background

When did you first hear of COVID?

Does COVID affect the mandate / jurisdiction of your organisation?

When did your organisation start to consider COVID as a policy issue / issue that needed dealing with?

- Was it viewed at first as an important issue or not?
  - What were the reasons for this?
  - Why did it take so long to respond? [OR alternative questioning if response was fast]
- How big of an issue is it for you now?

Do you understand what causes COVID and where it has come from?

- Where did you get your information about COVID from?
  - Was this officially communicated within your organisation.
  - Do you trust the information you have?
- Do you feel you know enough about the disease within your organisation?
  - What other information do you need?

Theme 2: The policy-level experience of COVID

How has COVID affected your organisation (e.g. in program delivery, policy development, existing policy areas etc)

- How big of an impact has it had?
  - Are you still able to deliver your programs?
- When did this start to be noticeable?
- How has this changed since the pandemic first came along?
  - Why?
  - Have other factors affected this (e.g. politics, weather, food prices)
- Has it affected the work that you do?
- Has it made your job more challenging?

Have the impacts of COVID or the policy response to COVID, affected certain regions or populations more than others?

- If yes or no, probe why, who, where etc

How big of an issue is COVID for [the study region of focus] and/or [Indigenous population of focus]: in general and for food systems in particular

- What makes them more or less at risk?
- Who are the most at risk during this pandemic?
- Have their concerns / needs affected influenced your organisation?
- Did you have migrant communities returning from cities or other places of work? Did your organization or region anticipate this type of migration?

Theme 3: COVID response

How serious an issue is responding to/dealing with COVID within your organisation?

- Has this changed since the pandemic came along?
- Do you prioritise certain regions, populations, sectors, in your response?
  - What about Indigenous peoples, rural areas

In what ways has your organisation responded to COVID / impacts of COVID policies etc for the study region and Indigenous peoples?

- How are you supporting communities?

How have Indigenous peoples [in general and/or the study region] been approached within the COVID response?

- Does your organization have information about how COVID is impacting indigenous communities? If yes, how does it get it? What information is it?
- Have there been consultations with IPs about COVID control or preventive strategies?
  - Has the consultation process changed since the pandemic started? Why?
    - What are the barriers to this?
  - In your opinion, do IPs have special needs, could you explain
- Have IP representatives been involved in decision-making?
  - Since when IPs were considered part of the priorities in the responses ? (Health/food security/ education/ communication/children/women/elders)
    - What are the barriers to this
- Have you communicated in local languages/dialects? Have you communicated with indigenous communities through local authorities or organizations, NGOs, radio...?
- Were the strategies from the government (lockdowns, etc) beneficial for IPs?
  - What measures worked well especially in addressing livelihood resilience?
  - What measures did not work as planned?

What has your organisation learned as the pandemic has progressed?

- Have these lessons been integrated into policy or decision making?

What do you think of how the government/authorities have handled COVID for [the study region] and [Indigenous peoples]?

- What could they have done differently?
- What constraints have they faced?

Now that a vaccine is a possibility in 2021, what challenges do you anticipate?

Theme 4: COVID in context

Do you think the response to COVID has made other problems worse for the study region and Indigenous peoples?

- E.g. for community resilience practices, livelihood practices
- E.g. around the current burden of ill-health
- E.g. around existing challenges facing food systems
- E.g. around existing challenges facing market access, land rights?

Has COVID created new opportunities for the study region and Indigenous peoples?

Have other factors made it more difficult/easier for you to respond and manage COVID?

Theme 5: Needs

What needs do you have in responding to COVID?

- More information, resources …….
- How can our project help
  - What is the best way of achieving this?

Theme 6: Evolution of the pandemic

- Did you hear about the new COVID-19 variants that have emerged over the pandemic? If yes, Could you mention it?
- What do you think would be the effects of the new variants for your organization?
- At present, how worried are you about the risk that COVID represents for your community? Could you explain
- Compared with the beginning of the pandemic, do you feel that at present you/your organisation are at a higher or lower risk of being affected by the COVID-19 pandemic? Could you explain why is this different?
- What do you think would be key lessons that this pandemic is leaving you/ your organisation to be better prepared for future pandemics?
- What would you do differently in the future if you hear that a new pandemic is emerging?
- Could you mention, what was the biggest challenge that you/your organisation had to face to respond to the COVID pandemic?

1. **Guide of the Community Observer base line interview**

Metadata:

For each observer interview document their:

- Community
- Age
- Gender
- Education
- Household size
- Occupation / livelihoods
- Length of time lived in the community
- Do you have access to a health facility in your community?
- How far is the nearest urban centre?
- What road infrastructure and transportation is available?

Theme 1: Epidemiology & COVID knowledge

Has anyone in your community had COVID?

- How do you know: Tested positive or perceived COVID, a doctor told, or have any symptomatology (please write the symptoms)?
- How common is it?
- Was there any fatality related to COVID in your community?
- Have those who recovered from COVID in your community experienced any lasting health impacts (disabilities)?

When was the first case reported in your community? And when were the most recent cases reported?

We could do a timeline with each observer to examine if/how cases have changed over time

(If you haven’t had any cases reported, how close to your community are there cases?)

Do you understand what causes COVID and where it has come from?

- Where did you get your information about COVID from?
  - Has this changed over the last 6 months
  - Do you trust the information you are getting?
- Do you feel you know enough about the disease?
- What steps are you taking to protect yourself from getting COVID?
- What other information do you need?

Theme 2: The lived experience of COVID

For many of us, COVID implies deeply feelings of concern, anxiety, confusion, and scariness. When you first heard of COVID how did you feel?

- Have your feelings changed since you first heard about COVID?

Describe the most significant change brought about by COVID to you/your community?

How else has COVID affected you and your community? (positive and negative impacts)

- Do impacts differ by:
  - Season
  - Occupation / livelihood
- Have you been able to maintain your traditional cultural practices

Has COVID affected your ability to access/produce your preferred food? (farming, fishing, hunting, foraging, herding, market access)

- Has it affected how, where, whom produces/collects/shares food?
- Has it made existing challenges of getting food more or less difficult?
- Who is most at risk and why?

Theme 3: Responding to COVID

Have the government/authorities acted on COVID? How?

- Do you/your community support these measures?
- Have they helped or hindered?
- What should they be doing extra?

What adjustments have been made in response to COVID by you/your community? (probe specific for food systems responses if required)

- How effective have they been?
- Do responses/effectiveness differ among community members (by age, gender, livelihood etc)
- Have there been any negative effects of the regulations themselves?

What factors have helped manage COVID/ helped make these responses effective? (probe for specific resilience/vulnerability factors that will be further outlined in our conceptual approach building on (*1-3*))

- Indigenous knowledge
- Strong links to place (e.g. sense of well-being and cultural strength derived from links to place, mobility, access to natural resources)
- Agency (e.g. local mobilisation to restrict access to communities)
- Institutions (e.g. role of government policy, role of traditional healers and traditional approaches to health, land tenure, community organizations)
- Collective action (e.g. sharing networks, local leadership, remittances)
- Learning (e.g. what have you learned about managing COVID, sources of learning)
- Individual and collective financial capitals

What factors have made managing COVID more difficult? Note: could have specific focus here around if/how COVID restrictions have affected these factors:

- Indigenous knowledge
- Strong links to place
- Agency
- Institutions (how easy has it been to access individual protective equipment - i.e. masks, sanitiser, etc.).
- Collective action
- Learning
- Individual and collective financial capitals

Theme 4: COVID in context

Have the existing health challenges you face made COVID more difficult?

- E.g. around the current burden of ill-health infectious (VIH, malaria, dengue, TB, snake bite, wild elephant attacks)
- E.g. around the current burden of ill-health chronic conditions (diabetes, hypertension, obesity, anaemia, other)
- E.g. around existing weaknesses in health systems

Has the impact of COVID been made more problematic by other factors you have limited control over locally?

- Government regulations
- Climatic and environmental factors: drought, wildfires, land degradation, biodiversity loss
- Marginalisation, land dispossession, and wider impacts of colonisation
- Social conflicts
- Individual and collective financial capital

Theme 5: Needs

What needs do you have in responding to COVID?

- e.g. More information, resources …….
- How can our project help?
  - What is the best way of achieving this?

Theme 6: Evolution of the pandemic

- Did you hear about the new COVID-19 variants that have emerged over the pandemic? If yes, Could you mention it?
- What do you think would be the effects of the new variants on your community / for your organization?
- At present, how worried are you about the risk that COVID represents for your community? Could you explain
- Compared with the beginning of the pandemic, do you feel that at present you/your community are at a higher or lower risk of being affected by the COVID-19 pandemic? Could you explain why is this different?
- What do you think would be key lessons that this pandemic is leaving you/ your community to be better prepared for future pandemics?
- What would you do differently in the future if you hear that a new pandemic is emerging?
- Could you mention, what was the biggest challenge that you/your community had to face to respond to the COVID pandemic?

1. **Guide of the Policy Observer follow-up interviews**

Key developments

- Record date of meeting
- Have there been any developments since the last time we met?
  - Status of COVID
  - Changes to government / authorities
  - Policy developments
  - Weather events
  - Food shortages
  - Diseases other than COVID
  - Other?

Theme 1: Background

Is COVID still an important issue for your organisation?

- Has this changed since the last time we met?
- What is the trend of COVID in your region? (e.g. around seasonality)

Since the last time you reported, are you and your organisation receiving information about COVID from different sources?

- What is the main source of information at the moment (e.g. government reports, scientists, Facebook, WhatsApp, word of mouth)
  - Do you trust the information you have?
- How is your organisation producing information about COVID? (Facebook, WhatsApp, word of mouth, Youtube**)**
  - Is this being targeted to communities in the study region? How?
  - Do you believe the community trust the information that you/government produces?

Is understanding on COVID improving?

Theme 2: The policy-level experience of COVID

Since the last time you reported, have there been any significant changes in how COVID has affected your organization (e.g. in program delivery, policy development, existing policy areas etc)

- Are you still able to deliver your programs?
- Has it affected the work that you do?
- Has it made your job more challenging?

Since the last time you reported, have the impacts of COVID or the policy response to COVID, affected certain regions or populations more than others?

- What makes them more or less at risk?
- Who are the most at risk during this pandemic?
- Did you have migrant communities returning from cities or other places of work? Did your organization or region anticipate this type of migration?

Theme 3: COVID response

Since the last time you reported, have there been any changes to how your organisation is responding to COVID / impacts of COVID policies?

Are there any new developments that need to be addressed by your organisation or government?

- Is the potential availability of a vaccine in 2021 affecting this?

Since the last time you reported, have there been any changes to how your organisation is approaching and consulting with Indigenous peoples?

- Are you consulting with IPs about COVID control or preventive strategies?
- Are you involving IP representatives in decision-making?
- Are you communicating in local languages/dialects?
- In what ways are the actions of your organisation benefiting people in the study regions?

Since the last time you reported, have you learnt any lessons on the pandemic and how to best respond?

What do you think about how the government / authorities are currently handling COVID [with regards IPs, the study region]?

- What specific needs do you currently have?

Is your organisation planning for how the pandemic might evolve in the future (e.g. second wave, or vaccine implementation?)

Theme 4: COVID in context

Are COVID policies, responses etc continuing to make other problems worse for the study region and Indigenous peoples?

- E.g. for community resilience practices
- E.g. around the current burden of ill-health
- E.g. around existing challenges facing food systems
- E.g. around existing challenges facing market access, land rights?

Since the last time you reported, has COVID created new opportunities for the the study region and Indigenous peoples?

Are other factors continuing to make it more difficult/easier for you to respond and manage COVID?

Theme 5: Triangulation

*The aim of this section is to generate questions building from the community diaries / stocktaking to develop policy observer insights on what is being reported locally. It is left open here as these questions and themes will very much depend on what is reported locally. An example could be: “the communities are saying the drought this year has made COVID much more problematic and has made getting government support very difficult. Can you comment on this?”* See note above for pertinent ethical considerations for this theme and the ethics WG document, as regional team need to be very careful to preserve the anonymity of individuals and/or communities when requested, and assess the risks of any disclosure.

Theme 6: Evolution of the pandemic

- Did you hear about the new COVID-19 variants that have emerged over the pandemic? If yes, Could you mention it?
- What do you think would be the effects of the new variants for your organization?
- At present, how worried are you about the risk that COVID represents for your community? Could you explain
- Compared with the beginning of the pandemic, do you feel that at present you/your organisation are at a higher or lower risk of being affected by the COVID-19 pandemic? Could you explain why is this different?
- What do you think would be key lessons that this pandemic is leaving you/ your organisation to be better prepared for future pandemics?
- What would you do differently in the future if you hear that a new pandemic is emerging?
- Could you mention, what was the biggest challenge that you/your organisation had to face to respond to the COVID pandemic?

1. **Guide of the Community Observer follow up interviews**

Key developments

*The aim here is to document any key developments, events, etc that have taken place since the previous meeting with the observer.*

- Record date of meeting
- Have there been any developments since the last time we met?
  - Policy developments
  - Weather events
  - Food shortages
  - Diseases other than COVID
  - Other?

Theme 1: Epidemiology & COVID knowledge

Have rates of COVID changed since the last time we met?

- Any fatalities/infections related to COVID?
- Are COVID cases/deaths increasing or decreasing?
- Has there been any local testing? and By who?
- Has there been a medical campaign or medical intervention in your community? What was it for? (e.g. vaccination, malaria screening)
- Has there been any type of campaign or intervention in your community? What was it for? (e,g, politicians, religious people, external aid)

Has your knowledge/community knowledge on COVID improved?

- Has understanding on the disease improved?
- Are you getting any additional advice from the authorities (government, indigenous organizations, NGOs, other actors)?
  - Do you trust the information you are getting?
- Do you feel you know enough about the disease?
- What steps have been taken recently to protect yourself/community from COVID?
- Have you heard about the new vaccine
  - What do you think of it
  - Do you think it will be available locally

Theme 2: The lived experience of COVID

Are you/people in the community still as (the reported feeling)/concerned about COVID as the last time we met?

- Why? What is different now?
- Has the potential new vaccine changed how you are thinking about COVID?

Since the last time we met, have there been any significant changes brought about by COVID to you/your community?

- Are you still able to maintain your traditional cultural practices? (e.g. cultural dances, communal farming/gardening...)
- Are you still able to access/produce/share your preferred food? (farming, fishing, hunting, foraging, herding, market access)
- Is COVID still affecting how, where, whom produces/collects/shares food?

Theme 3: Responding to COVID

Are the measures being adopted by the government/authorities that you described last time effective? (e.g. Lockdowns, curfew, limitations on gatherings, food distributions etc)

- Is there still support for them?

Have there been any developments in how the government/authorities are responding to COVID since we last met?

- How were these communicated?
- Do you/your community support these measures? why?/why not?
- Are they listening to local needs and concerns?
- Do you think they will be effective? Why?

Are you/your community still utilising the same responses you described last time?

- Are they effective?
- What makes them effective / not effective?
- Is everyone utilising these responses?

Are you responding in new ways since we last met?

- Why are you responding in this way?
- Do you think it will be effective?

What factors are helping manage COVID? (probe for specific resilience/vulnerability factors. Potential to focus on those identified in the stocktaking and/or previous interviews)

- Indigenous knowledge
- Strong links to place (e.g. sense of well-being and cultural strength derived from links to place, mobility, access to natural resources)
- Agency (e.g. local mobilisation to restrict access to communities)
- Institutions (e.g. role of govn policy, role of traditional healers and traditional approaches to health, land tenure, access to health care services, access to health insurance...)
- Collective action (e.g. sharing networks, local leadership, remittances)
- Learning (e.g. what have you learned about managing COVID)
- Other

What factors are making managing COVID more difficult? Note: could have specific focus here around if/how COVID restrictions have affected these factors:

- Indigenous knowledge (e.g. generational/gendered differences/lack of application)
- Strong links to place (e.g. restrictions compromising ability to practice traditional activities)
- Agency (e.g. ability to respond is constrained, limits to livelihood diversification)
- Institutions (e.g. how easy has it been to access personal protective equipment - i.e. masks, sanitiser, etc.)
- Collective action (e.g. have remittances declined, have sharing networks been affected by reduced traditional food access, lack of agreement on what needs to be done)
- Learning (e.g., limited learning opportunities)
- Other

Theme 4: COVID in context

Are existing health challenges continuing to make COVID more difficult?

Has the impact of COVID been made more problematic by other factors you have limited control over locally?

- Government regulations
- Climatic and environmental factors: drought, wildfires, land degradation, flooding, landslides, biodiversity loss
- Marginalisation and land dispossession

Theme 5: Needs

What needs do you have in responding to COVID?

- e.g. More information, resources …….
- How can our project help?
  - What is the best way of achieving this?

Theme 6: Evolution of the pandemic

*The aim of this section is to explore the new variants(s) of COVID-19 perceptions of risk, the differences between this variant and COVID-19’s original emergence, and how is this leading to learning.*

- Did you hear about the new COVID-19 variants that have emerged over the pandemic? If yes, Could you mention it?
- What do you think would be the effects of the new variants on your community / for your organization?
- At present, how worried are you about the risk that COVID represents for your community? Could you explain
- Compared with the beginning of the pandemic, do you feel that at present you/your community are at a higher or lower risk of being affected by the COVID-19 pandemic? Could you explain why is this different?
- What do you think would be key lessons that this pandemic is leaving you/ your community to be better prepared for future pandemics?
- What would you do differently in the future if you hear that a new pandemic is emerging?
- Could you mention what was the biggest challenge that you/your community had to face to respond to the COVID-19 pandemic?

**Appendix B. List of national and regional policy documents**

| Policy | Date | Scope | Leading government sector | Detail of the policy | N° of Policy document |
| --- | --- | --- | --- | --- | --- |
| Emergency declaration | 11/03/20 | National | Central government | National sanitary emergency for 90 days and prevention and control measures for COVID-19 | DS N° 008-2020-SA |
| Health budget | 11/03/20 | National | Ministry of Health | The Ministry of Health provides a budget to regional governments to guarantee the continuity of health services during the pandemic | DU Nº 025-2020 |
| National Quarantine | 15/03/20 | National | Central government | National emergency for 15 days and national quarantine (reduce mobilization) | DS Nº 044-2020-PCM |
| Food and cash aid | 16/03/20 | National | Ministry of Development and Social Inclusion | Measures to reduce the economic impact of COVID-19 through food and cash aid for poor households | DU Nº 027-2020 |
| Health budget | 18/03/20 | National | Ministry of Health | The Ministry of Health provides a budget for regional governments to guarantee health services during the pandemic | RM N° 96-2020-MINSA |
| National Quarantine | 15/03/20 | National | Central government | National quarantine is announced, with mandatory lockdowns for certain hours | DS Nº 046-2020-PCM |
| Food and cash aid | 27/03/20 | National | Ministry of Labor and Employment Promotion | Measures to reduce the economic impact of COVID-19, through food and cash aid for vulnerable independent workers | DU Nº 033-2020 |
| Health guide for COVID-19 | 30/03/20 | National | Ministry of Health | Health normative for the prevention of COVID-19 and the treatment of people infected by COVID-19 | RM N° 139-2020/MINSA |
| Cash aid | 07/04/20 | National | Ministry of Development and Social Inclusion | Social programs transfer cash aids in advance to beneficiaries | DS N° 006-2020-MIDIS |
| Cash aid | 10/04/20 | National | Ministry of Labor and Employment Promotion | Measures to reduce the economic impact of COVID-19, through cash aid for vulnerable households with independent workers | DU Nº 036-2020 |
| Health guide for COVID-19 | 13/04/20 | National | Ministry of Health | Health normative for the prevention, diagnosis and treatment of COVID-19 | RM N° 193-2020-MINSA |
| Cash aid | 19/04/20 | National | Ministry of Development and Social Inclusion | Measures to reduce the economic impact of COVID-19, through cash aid for rural households in conditions of poverty | DU Nº 042-2020 |
| Health guide for COVID-19 | 20/04/20 | National | Ministry of Health | Modification of the health normative for the prevention and diagnosis of COVID-19 and the treatment of people infected by COVID-19 | RM N° 209-2020/MINSA |
| Cash aid | 21/04/20 | National | Ministry of Development and Social Inclusion | Measures to reduce the economic impact of COVID-19, through cash aid for poor households | DU Nº 044-2020 |
| Health guide for COVID-19 | 29/04/20 | National | Ministry of Health | Modification of the health normative for the prevention and diagnosis of COVID-19 and the treatment of people infected by COVID-19 | RM N° 240-2020/MINSA |
| Food aid | 30/04/20 | National | Central government | National School Program of Food (Qaliwarma) provides food to people in a situation of vulnerability, in the health emergency declared by COVID-19 | DL Nº 1472 |
| Indigenous Peoples | 04/06/20 | National | Ministry of Culture | Guidelines for the delivery of food aid for Indigenous people | DS N 008-2020-MC |
| Cash aid | 05/05/20 | National | Ministry of Development and Social Inclusion | Measures to reduce the economic impact of COVID-19, through cash aid for poor households | DU Nº 052 -2020 |
| Health guide for COVID-19 | 08/05/20 | National | Ministry of Health | Modification of the health normative for the prevention and diagnosis of COVID-19 and the treatment of people infected by COVID-19 | RM N° 270-2020/MINSA |
| Indigenous Peoples | 10/05/20 | National | Central government | National policy for the protection of Indigenous people during the COVID-19 health emergency | DL Nº 1489 |
| Health guide for COVID-19 | 06/05/20 | National | Ministry of Health | Health normative of the management of people infected by COVID-19 in critical care areas | RM N° 254-2020/MINSA |
| Indigenous Peoples | 21/05/20 | National | Ministry of Health | Plan of intervention of Health of Ministery for amazon Indigenous and rural communities | RM N 308-2020-MINSA |
| Health guide for COVID-19 | 24/05/20 | National | Ministry of Health | Health normative about the list of essential goods for the management and treatment of COVID-19 | RM N° 315-2020/MINSA |
| Stadistics | 29/05/20 | National | Ministry of Health | Guide for the incorporation of the ethnic variable in administrative records of COVID-19 infected population | DS N° 005-2020- MC |
| Indigenous Peoples | 06/06/20 | Regional/ Junin | Regional government of Junin | Creation of COVID-19 command in Junin region | RER N° 187-2020-GRJ-GR |
| Indigenous Peoples | 08/06/20 | Regional/ Loreto | Regional government of Loreto | Creation of COVID-19 command in Loreto region | RER N° 155-2020-GRL-GR |
| Health guide for COVID-19 | 09/06/20 | National | Ministry of Health | Health normative of the management of people infected by COVID-19 | RM N° 375-2020/MINSA |
| Indigenous Peoples | 12/06/20 | National | Ministry of Health | Technical document for the formation and operation of the COVID-19 Indigenous commands | RM N.° 386-2020-MINSA |
| Indigenous Peoples | 18/06/20 | National | Ministry of Culture | Temporal Multisectoral Commission to follow-up actions for the protection of indigenous people during the pandemic | RS N° 005-2020-MC |
| Health guide for COVID-19 | 19/06/20 | National | Ministry of Health | Modification of the health normative about the list of essential goods for the management and treatment of COVID-19 | RM N° 419-2020/MINSA |
| Indigenous Peoples | 01/07/20 | National | Ministry of Health | Guidelines for the prevention of respiratory infections and COVID-19 in indigenous peoples in isolation or initial contact | RM N.° 451-2020-MINSA |
| Indigenous Peoples | 21/07/20 | National | Ministry of Health | Management of COVID-19 deaths corpse among Indigenous peoples | RM N.° 512-2020-MINSA |
| Indigenous Peoples | 30/07/20 | National | Ministry of Culture | Normative for the supervision of COVID-19 cases in Indigenous peoples | DS N° 010-2020- MC |
| Cash aid | 20/08/20 | National | Ministry of Development and Social Inclusion | Measures to reduce the economic impact of COVID-19, through cash aid for poor households | DU Nº 098-2020 |
| Indigenous Peoples | 30/08/20 | National | Ministry of Culture | Guide for areas where there is a presence of Indigenous peoples in isolation or initial contact, during the COVID-19 health emergency | DS Nº 014-2020-MC |
| Health guide for COVID-19 | 12/10/20 | National | Ministry of Health | Health normative of the management of people infected by COVID-19 in hospitalisation | RM N° 839-2020/MINSA |
| Vaccination | 16/10/20 | National | Ministry of Health | Approbation of national COVID-19 vaccination plan | RM N.° 848-2020-MINSA |
| Health guide for COVID-19 | 20/11/20 | National | Ministry of Health | Modification of the health normative of the management of people infected by COVID-19 | RM N° 947-2020/MINSA |
| Cash aid | 30/01/21 | National | Ministry of Development and Social Inclusion | Measures to reduce the economic impact of COVID-19, through cash aid for poor households | DU Nº 010-2021 |
| Indigenous Peoples | 18/04/21 | National | Ministry of Health | Modification of the document for the formation and operation of the COVID-19 Indigenous commands | RM N° 506-2021/MINSA |
| Health guide for COVID-19 | 07/07/21 | National | Ministry of Health | Modification of the health normative of the management of people infected by COVID-19 | RM N° 834-2021/MINSA |
| Health guide for COVID-19 | 26/07/21 | National | Ministry of Health | Modification of the health normative of the management of people infected by COVID-19 | RM Nº 938-2021/MINSA |
| Cash aid | 23/08/21 | National | Ministry of Development and Social Inclusion | Measures to reduce the economic impact of COVID-19, through cash aid for poor households | DU Nº 080-2021 |
